# Anti-Ri paraneoplastic neurological syndrome presenting with bilateral cranial nerve VI palsy and jaw dystonia—a distinctive syndrome within the anti-Ri spectrum? Case report and literature review

**DOI:** 10.1007/s10354-023-01006-8

**Published:** 2023-03-03

**Authors:** Elisabeth Freydl, Alexander Tinchon, Katrin Blauensteiner, Stefan Oberndorfer

**Affiliations:** 1 Karl Landsteiner University of Health Sciences, Dr.-Karl-Dorrek-Straße 30, 3500 Krems, Austria; 2 Department of Neurology, University Hospital St. Pölten, Dunant-Platz 1, 3100 St. Pölten, Austria; 3 Karl Landsteiner Institute of Clinical Neurology and Neuropsychology, University Hospital St. Pölten, Dunant-Platz 1, 3100 St. Pölten, Austria; 4 Neurological Department, Clinic of Floridsdorf, Brünner Str. 68, 1200 Vienna, Austria

**Keywords:** Onconeuronal antibodies, Antineuronal antibodies, Surface antigens, Intracellular antigens, Oculomotor disorder, Onkoneuronale Antikörper, Antineuronale Antikörper, Oberflächenantigene, Intrazelluläre Antigene, Optomotorikstörung

## Abstract

**Objective:**

Paraneoplastic neurological syndromes (PNS) are rare disorders associated with various onconeuronal antibodies. Anti-Ri antibodies (ANNA-2) are typically found in patients with opsoclonus myoclonus syndrome (OMS) and ataxia.

**Case report:**

We present an anti-Ri antibody-positive 77-year-old woman with subacute progressive bilateral cranial nerve VI palsy, gait disturbance and jaw dystonia. MRI of the brain showed hyperintense signals on T_2_ bitemporal without contrast enhancement. Cerebrospinal fluid (CSF) examination exhibited mild pleocytosis of 13 cells/µl and positive oligoclonal bands. CSF was overall inconspicuous for a malignant or inflammatory etiology. Immunofluorescence analysis revealed anti-Ri antibodies in both serum and CSF. Subsequent diagnostic work up resulted in a newly diagnosed ductal carcinoma of the right breast. PNS in this case partially responded to the anti-tumor therapy.

**Conclusion:**

This case shows similarities with recently published anti-Ri syndromes, which might form a distinct triad within the anti-Ri spectrum.

## Abstract (de)

**Hintergrund:**

Paraneoplastische neurologische Syndrome (PNS) sind seltene Erkrankungen, die mit zahlreichen onkoneuronalen Antikörpern assoziiert sein können. Anti-Ri-Antikörper (ANNA-2) werden im Regelfall bei Patienten mit Opsoklonus-Myoklonus-Syndrom (OMS) und Ataxie gefunden. In der vorliegenden Arbeit wird der Fall einer 77-jährigen Frau mit einer subakuten beidseitigen N.-abducens-Parese, Gangstörung und mandibulärer Dystonie präsentiert.

**Fallbericht:**

In der Anamnese war Brustkrebs vor 11 Jahren und ein bereits reseziertes Angiosarkom vor 6 Monaten zu erheben. In der Magnetresonanztomographie (MRT) waren bitemporale T2-Signalhyperintensitäten ohne Kontrastmittelaufnahme erkennbar. Die Lumbalpunktion erbrachte eine leichtgradige Pleozytose mit 13 Zellen/µl und positive oligoklonale Banden. Es fanden sich keine Hinweise auf eine maligne oder entzündliche Ätiologie im Liquor. In der Immunfluoreszenz konnten Anti-Ri-Antikörper in Serum und Liquor nachgewiesen werden. Die anschließende Tumorsuche erbrachte ein neu aufgetretenes duktales Mammakarzinom. Das PNS sprach im vorliegenden Fall teilweise auf die Anti-Tumor-Therapie an.

**Schlussfolgerung:**

Dieser seltene Fall demonstriert die klinische Variabilität von paraneoplastischen Syndromen, die ein umfassendes Tumorscreening nach sich ziehen sollten. Wiederholte Fallberichte sehr ähnlicher klinischer Manifestationen lassen ein eigenständiges Syndrom innerhalb des Anti-Ri-Spektrums vermuten.

## Introduction

Classical PNS are associated with well-characterized autoantibodies against intracellular antigens and occur mainly in elderly people [1]. They are frequently associated with cancer and commonly precede a tumor diagnosis. Approximately 0.01% of cancer patients develop PNS [2]. The course of disease is usually monophasic and characterized by a poor treatment response to steroids or immunoglobulins, with often irreversible neurological deficits [3, 4]. A definite PNS can be diagnosed if the classical neurological syndrome is associated with cancer, or if the cancer is diagnosed within 5 years after onset of the neurological syndrome [3]. Also atypical neurological syndromes which improve after cancer therapy or which are positive for onconeuronal antibodies support the diagnosis of PNS [5].

Anti-Ri antibody-associated PNS typically presents with OMS and ataxia, although other clinical syndromes have been reported [5–8]. This rare paraneoplastic syndrome is more common in women and occurs with SCLC and breast cancer [5, 9–11].

We report a distinct clinical presentation of anti-Ri syndrome in a patient with previously known cancer. Due to the suspected diagnosis of PNS, screening for occult cancer was initiated and exhibited a newly diagnosed second malignancy.

## Case report

A 77-year-old woman initially presented with double vision, vertigo, weight loss and unsteady gait persisting for several weeks. Three months before admission, similar fluctuating symptoms without visual impairment had been reported. The patient had undergone breast surgery with consecutive radiation because of breast cancer 11 years previously. Additionally, an angiosarcoma of the breast had been diagnosed and resected 6 months before admission.

Initial neurological examination on admission revealed an isolated oculomotor disorder with slightly bilateral adduction in primary eye position at rest and isolated abduction weakness affecting both eyes, clinically suspicious for a bilateral cranial nerve VI palsy. At this time, no other clinical abnormalities were found: motor and sensory function were inconspicuous, there was no evidence of trunk or limb ataxia, muscle tone and tendon reflexes were normal. The patient was admitted for further diagnostic work-up. In the ophthalmological assessment, the pupils were of equal size and reactive to light. The ocular motility examination confirmed bilateral weakness of abduction (right eye > left eye) and consecutive double vision without further signs of neurological involvement.

**Fig. 1:**
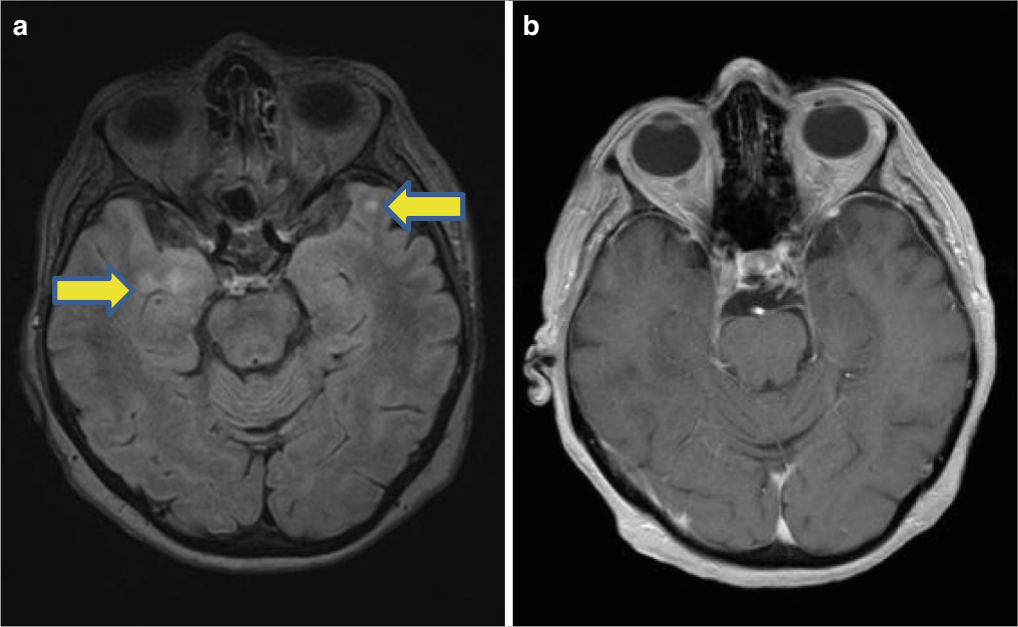
**a** Cerebral MRI T2-weighted (fluid-attenuated inversion recovery); *yellow arrows* indicate bitemporal hyperintense signals. **b** Cerebral MRI T1-weighted + gadolinium: no contrast enhancement

Compared with a reference MRI of the brain acquired 3 months before admission, subsequent MRIs within a timeframe of 2 weeks after admission showed increased bitemporal signal activity on T_2_ without gadolinium enhancement, morphologically consistent with limbic encephalitis but without any evidence of brainstem encephalitis or paraneoplastic cerebellar degeneration (see Fig. 1). MRI of the spine revealed no relevant radiological features. A distinct reason for the gait disturbance could be evaluated neither clinically, nor by imaging or lumbar puncture. Neuropsychological testing, including global cognitive function, was unremarkable.

Basic laboratory testing showed only non-specific findings, with elevated ESR and bilirubin and reduced albumin, but no particular signs of diabetes, which also causes ophthalmoplegia. Comprehensive tests looking for an infectious origin (HIV, CMV, HSV, VZV, EBV, TB, tetanus, borreliosis, tick-borne encephalitis, mycoplasma, and syphilis) remained negative. Furthermore, ACE and β2-microglobulin were within the normal ranges.

CSF examination showed a slightly elevated cell count of 13 cells/µl and a mildly elevated protein level at 54 mg/dl (laboratory reference: 15–45 mg/dl). Oligoclonal bands were detected in the CSF, but there was no evidence of malignant cells. Immunofluorescence analysis revealed anti-Ri antibodies in both serum and CSF (1:10,000).

Ten days after admission, the patient developed painless jaw dystonia in the form of an oromandibular lockjaw. At this time, the bilateral abduction weakness on both eyes deteriorated, but again with no evidence of a distinct cerebral oculomotor syndrome such as internuclear ophthalmoplegia. With a view to differential diagnoses, immunosuppression was initiated with methylprednisolone 1 g per day for 5 days right after diagnosis. Due to an insufficient clinical response, intravenous immunoglobulins with 30 g per day for 5 days were subsequently administered.

Chest and abdominal CTs were obtained and a new tumor of the right breast, highly suspicious for malignant breast cancer, was found. Additionally, FDG-PET demonstrated a higher tracer uptake in this region. Higher uptake was also noted in some subclavicular and retroclavicular lymph nodes. With an ultrasound-guided biopsy, a new ductal carcinoma of the breast was finally diagnosed.

Subsequently, the patient underwent quadrantectomy of the right breast, and systemic treatment with anastrozole was initiated about 6 weeks after admission. In the further course of disease, continuous clinical improvement of the bilateral cranial nerve VI palsy and gait disturbance was observed and the patient was discharged. To reduce the persistent jaw dystonia and enable the patient to eat and speak, botulinum toxin was applied to the masseter muscles with 30 MU on each side in an ambulatory setting. Due to a clinical improvement 3 months after the first application, botulinum toxin was administered again with 50 MU each side.

Five months after the initial presentation, the jaw dystonia had improved and our patient had regained weight. Bilateral cranial nerve VI palsy had also improved and double vision diminished. Due to the good response to the anti-tumor treatment with anastrozole, steroids were tapered down and finally discontinued. One month later she was rehospitalized due to melena. While gastroscopy was unremarkable, laboratory examination showed decreased red blood cells and platelets, thus requiring transfusions. Subsequently, the patient suffered from respiratory insufficiency caused by hemothorax and underwent artificial respiration. In a CT of the chest, tumor progression with metastases in lung, liver, supra- and infraclavicular lymph nodes and bones was found.

Taking the progressive tumor disease into account, despite a clinical improvement of PNS, therapeutic retreat and supportive care were established and the patient died shortly thereafter.

## Discussion

Anti-Ri PNS is usually associated with OMS as well as with ataxia. The current case is an unusual and rare presentation of anti-Ri syndrome with gait disturbance, bilateral cranial nerve VI palsy and oromandibular dystonia. Atypical clinical presentations of anti-Ri-positive syndromes such as cranial nerve palsies and other oculomotor abnormalities, jaw dystonia and laryngospasms have been reported in rare cases [5, 9, 12, 13].

The diagnosis of anti-Ri syndrome usually follows the updated diagnostic criteria for PNS of the international PNS-Care panel. It is defined as neurologic disorder that (1) can affect any part of the nervous system, often presenting with stereotyped clinical manifestations; (2) occur in association with cancer and (3) have an immune-mediated pathogenesis that is supported by the frequent presence of specific neuronal antibodies [14]. See Table 1 for an overview of anti-Ri syndromes with different clinical features and underlying tumor diseases. Even in the case of negative antibody results, it should be kept in mind that in classical PNS, well-characterized onconeuronal antibodies can only be detected in 25% of clinically preselected patients [2]. However, their appearance strongly suggests an underlying malignant disease in the present or in the future. In contrast, partially characterized antibodies provide limited clinical experience and have weaker associations with distinct tumor entities. Nonetheless, diagnostic work-up largely matches with cases of well-characterized antibodies and also focusses on tumor detection. Onconeuronal antibodies have to be distinguished from surface antibodies in several aspects. First, detection of surface targets is mainly realized by cell-based assays (e.g., immunohistochemistry), while ELISA and immunoblotting are additionally used for intracellular proteins. Second, the response to treatment is favorable in most surface antibody-related diseases, while it is rather poor in tumor-associated cases with proven onconeuronal antibodies [15]. Third, there should be awareness for false-positive as well as false-negative antibody results. For example, when using radioimmune assays, VGKC antibodies exhibit a high false test rate [15]. In contrast, negative antibody results do not exclude the possibility of PNS in clinically suspicious cases. However, a tumor diagnosis in such cases should also be considered as a coincident event [14].

**Table 1:** Overview of anti-Ri syndromes with different clinical features and underlying tumor diseases

Case study	Author	Journal and date of publication	Underlying malignancy
Pearls & Oysters: gait instability, jaw dystonia and horizontal diplopia in a woman with anti-Ri antibodies and breast cancer	Alkabie S. et al. [24]	*Neurolog*y 2022	Breast cancer
Anti-Ri antibody-associated paraneoplastic syndrome in a man with breast cancer showing a reversible pontine lesion on MRI	Kim H. et al. [16]	*J Clin Neurol.* 2009	Breast cancer
Anti-Ri-associated paraneoplastic cerebellar degeneration. Report of a case and revision of the literature	Mancuso M. et al. [8]	*Arch Ital Biol* 2011	SCLC
Anti-Ri-associated paraneoplastic neurological syndrome: initial symptom of breast cancer with HER2 overexpression and treatment by dual HER2 blockade	Olmez O. F. et al. [10]	*J Oncol Pharm Pract.* 2019	Breast cancer
Clinical reasoning: a 43-year-old man with subacute onset of vision disturbances, jaw spasms and balance and sleep difficulties	Orozco E. et al. [25]	*Neurology* 2022	No tumor found
Paraneoplastic jaw dystonia and laryngospasm with antineuronal nuclear autoantibody type 2 (anti-Ri)	Pittock SJ. et al. [9]	*Arch Neurol.* 2010	Breast cancer Sweat gland angiosarcoma Uterine cervical carcinoma
Anti-Ri-associated paraneoplastic ophthalmoplegia ataxia syndrome in a woman with breast cancer: a case report and review of the literature	Sena G. et al. [11]	*J Med Case Rep.* 2020	Breast cancer
Anti-Ri antibody-associated small cell lung carcinoma	O’Leary CG. et al. [26]	*Ir J Med Sci.* 2017	SCLC

The diagnosis of anti-Ri syndrome can further be assisted by various examinations. An MRI of the brain may show hyperintense T2 lesions, mainly affecting the brainstem, with or without gadolinium enhancement, which can regress after treatment [16]. Only half of the patients with anti-Ri syndrome show MRI signal abnormalities affecting various brain regions like the insular cortex, pons, and the hemispheric white matter [9]. The CSF is altered in the majority of patients, with intrathecal immunoglobulin production as the most common finding, including slightly elevated leukocytes and protein [3, 16].

PNS commonly precedes a tumor diagnosis; thus, screening for an unknown malignancy is mandatory. In addition to clinical and radiological tests, FDG-PET is recommended in some cases [17]. In patients with PNS and a former history of cancer, an additional new malignancy should be considered. If no tumor can be found, follow-up tests should be performed every 6 months for up to 4 years, with the exception of LEMS, in which screening for 2 years is sufficient [18]. In almost 80% of patients with PNS, the tumor is diagnosed within 4–6 months after the presentation of paraneoplastic syndrome [19].

Beside the classical clinical presentation, also atypical subacute neurological signs and symptoms should be considered as possible PNS. For example, OMS was believed to be the stereotyped manifestation of a paraneoplastic anti-Ri syndrome. Conversely, Simard et al. argued that only 25% of a French cohort were affected by OMS. They discuss anti-Ri syndrome as a progressive, multisystemic neurological disease with a cerebellar syndrome as the primary manifestation, being accompanied by various other symptoms [20].

The exact pathomechanism for distinct clinical symptoms in anti-Ri syndromes remains unclear. For the very rare manifestation of jaw dystonia, for example, a specific peptide-directed attack of upregulated major histocompatibility complex class 1 molecules bearing ANNA‑2 peptide fragments has been assumed [9]. In general, most paraneoplastic syndromes can be considered immunomediated reactions to the central or peripheral nervous system, either by antibodies or cytotoxic T cell-related mechanisms [21].

No larger prospective trials regarding standardized treatment for PNS are available. It seems that early anticancer therapy can improve neurological signs and symptoms, as seen in our and other reported cases [9, 13, 15]. Additionally, immunosuppressive methods like intravenous steroids or immunoglobulins can be applied. It should be mentioned that there is no evidence for any use of immunosuppressive regimens in patients with onconeuronal antibodies [22]. From a pathophysiological point of view, onconeuronal antibodies cannot be sufficiently removed by plasmapheresis, as they are located intracellularly. Nonetheless, some of these therapeutic approaches can be observed in clinical practice, as they are usually applied in doubt, before the definite diagnosis has been made. Steroids may be administered orally and tapered down, depending on the neurological symptoms. In some cases, plasmapheresis combined with cyclophosphamide has been successful [2, 23]. In some cases, like the current patient, additional symptomatic treatment for local muscle relaxation can also be required.

Two very recent publications, both published in *Neurology* in 2022, and a nationwide French case series of 36 patients stretching over 20 years have also reported on anti-Ri-associated clinical cases.

Alkabie et al. described a very similar case study, albeit divergent in some aspects. In terms of signs and symptoms, common findings were weight loss, jaw dystonia and diplopia [24]. The diagnostic work-up of both cases revealed T2 hyperintensities on MRI without gadolinium enhancement, positive oligoclonal bands in CSF, anti-Ri antibodies in serum and CSF, as well as underlying breast cancer. The main difference was the absence of limb weakness and stiffness in our case, which could be expected in a patient suffering from unsteady gait. Another difference was the well-documented bilateral cranial nerve VI palsy in our patient, whereas diplopia in Alkabie’s case could not clearly be assigned to a certain oculomotor disorder. Nonetheless, the suggested clinical triad comprising gait instability, jaw dystonia, and horizontal gaze paresis essentially also applies to our case [24].

Orozco et al. reported on an anti-Ri brainstem syndrome with a multiphase disease course, resulting in a severe manifestation with horizontal gaze ophthalmoplegia, jaw-closing dystonia, and other typical brainstem symptoms such as vertical nystagmus, dysarthria and dysphagia. Gait incoordination was also reported in this patient in early disease progression. Another finding common with our case were the positive oligoclonal bands in CSF as well as T2 hyperintensities in cerebral MRI. The initially suspected germ cell tumor in this patient could not be confirmed in the histological work-up [25].

Simard et al. published a comprehensive, 20-year retrospective case series of 36 patients and a literature review of anti-Ri syndromes in *Neurology* in 2020 [20]. The authors found four patterns of disease onset, comprising cerebellar symptoms, isolated tremor, oculomotor disorders and other symptoms. In the plateau phase of the disease, which was reached at a median of 12 weeks after onset, two groups emerged: those with cerebellar symptoms (67%) and those without (33%). Oculomotor disturbances were present in both groups and comprised 6 patients. While all of these patients suffered from diplopia, 4/6 patients had objective deficits (ophthalmoplegia: 2, internuclear ophthalmoplegia: 1, cranial nerve III palsy: 1). Jaw dystonia was also described as an infrequent symptom in 2 patients of this French cohort. Oligoclonal bands in CSF were found in 80%, whereas T2 hyperintensities could only be detected in 18% of patients in different brain regions. A cancer diagnosis was reported in the majority of patients (92%), with breast cancer being the most common underlying malignancy (61%).

Summarizing the above, recent publications have led to an updated view of anti-Ri syndromes. There is increasing evidence to indicate that what was predominantly designated as OMS and ataxia in the past can now be described across a more complex, multisystemic paraneoplastic spectrum. It is not just that this syndrome initially often presents with a combination of other neurological symptoms, but also that its appearance can change during the course of disease [20]. Our patient joins a series of recent reports that focus on a combination of gait disturbance, oculomotor disorders and jaw dystonia. This raises suspicion that such a triad may contribute to a distinctive syndrome within the anti-Ri spectrum. To our knowledge, this is also the first case of a bilateral cranial nerve VI palsy as an oculomotor manifestation of anti-Ri syndrome. However, a finely differentiated description of neuroophthalmological syndromes may not have always been consistently reported in the past.

In conclusion, anti-Ri-associated PNS can clinically appear with varying neurological signs and symptoms. Therefore, positive onconeuronal antibody findings should be considered potentially paraneoplastic, with a diagnostic focus on comprehensive tumor screening. The literature suggests that a combination of oculomotor abnormalities, gait disturbance and jaw dystonia may represent a distinctive paraneoplastic anti-Ri syndrome.

## Abbreviations

ACE: Angiotensin-converting-enzyme
CMV: Cytomegalovirus
CT: Computed tomography
EBV: Epstein-Barr virus
ELISA: Enzyme linked immunosorbent assay
ESR: Erythrocyte sedimentation rate
FDG-PET: F-Fluorodeoxyglucose positron-emission tomography
HIV: Human immunodeficiency virus
HSV: Herpes simplex virus
IVIG: Intravenous immunoglobulins
LEMS: Lambert–Eaton myasthenic syndrome
MRI: Magnetic resonance imaging
PNS: Paraneoplastic neurological syndromes
SCLC: Small cell lung cancer
TB: Tuberculosis
VZV: Varicella zoster virus
